# The Pathophysiological Relevance of the iNKT Cell/Mononuclear Phagocyte Crosstalk in Tissues

**DOI:** 10.3389/fimmu.2018.02375

**Published:** 2018-10-12

**Authors:** Filippo Cortesi, Gloria Delfanti, Giulia Casorati, Paolo Dellabona

**Affiliations:** ^1^Experimental Immunology Unit, Division of Immunology, Transplantation and Infectious Diseases, IRCCS San Raffaele Scientific Institute, Milan, Italy; ^2^Università Vita-Salute San Raffaele, Milan, Italy

**Keywords:** NKT cells, CD1d, monocytes, macrophages, DC, microenvironment

## Abstract

CD1d-restricted Natural Killer T (NKT) cells are regarded as sentinels of tissue integrity by sensing local cell stress and damage. This occurs via recognition of CD1d-restricted lipid antigens, generated by stress-related metabolic changes, and stimulation by inflammatory cytokines, such as IL-12 and IL-18. Increasing evidence suggest that this occurs mainly upon NKT cell interaction with CD1d-expressing cells of the Mononuclear Phagocytic System, i.e., monocytes, macrophages and DCs, which patrol parenchymatous organs and mucosae to maintain tissue homeostasis and immune surveillance. In this review, we discuss critical examples of this crosstalk, presenting the known underlying mechanisms and their effects on both cell types and the environment, and suggest that the interaction with CD1d-expressing mononuclear phagocytes in tissues is the fundamental job of NKT cells.

## Introduction

Natural Killer T (NKT) cells are a subset of T lymphocytes with innate-like functions characterized by the ability of recognizing lipid antigens presented by the major histocompatibility complex (MHC)-related molecule CD1d (1). NKT cells can be divided into two groups according on their TCR usage. Type I or invariant (i)NKT cells express a TCR made by the invariant rearrangement Vα14-Jα18 (*TRAV11*–*TRAJ18*) in mice, and the orthologous Vα24-Jα18 (*TRAV10*–*TRAJ18*) in humans, paired with diverse β-chains that utilize a restricted set of Vβ genes (2, 3). Type II NKT cells express different, yet poorly diverse, TCRs other than the semi-invariant Vα14/Vα24 one (4, 5).This review will focus on iNKT cells, the most represented and best characterized subset.

iNKT cells are endowed with a constitutive (i.e., innate) effector-memory phenotype: unlike mainstream MHC-restricted T cells, they rapidly produce large amounts of inflammatory and regulatory cytokines and chemokines upon activation without prior antigen sensitization (6, 7). This innate reactivity, together with their primary localization in tissues, makes iNKT cells effective sentinels of tissue integrity. Mouse and human iNKT cells have been found in lung, intestinal and urogenital mucosae, skin, fat, parenchymatous organs, as well as secondary lymphoid organs. There, they respond to two main types of stimuli, resulting from cell damage and inflammation induced upon pathological processes, namely: (i). signaling from pro-inflammatory cytokines, particularly IL-12 and IL-18 (8, 9); (ii). recognition of microbial or autologous (self) agonist lipids presented by CD1d, which derive from infecting pathogens and from biosynthetic pathways upregulated by stress in immune cells, respectively (10–15). A critical aspect of this function, supported by increasing body of evidence, seems to be represented by the highly regulated crosstalk between iNKT cells and a broad range of CD1d-expressing cell populations of the mononuclear phagocyte system (MPS), represented by monocytes, macrophages, and DCs (10, 14–19). Owed to its extensive diversity and plasticity, the MPS plays essential functions in the organism, including tissue maintenance and healing, innate immune responses and pathogen clearance, and the induction of adaptive immune responses (20–22). Importantly, the cells of the MPS express CD1d in mice and humans and are strategically positioned in tissues to sense stress and convey it to iNKT cells to coordinate a rapid reaction against it. Through these bidirectional interactions with MPS, iNKT cells rapidly modulate the local microenvironment for an immediate tissue reaction, concurrently helping the induction of subsequent adaptive immune responses. In this review, we propose that the interaction with CD1d-expressing MPS in tissues is the fundamental job of iNKT cells, and we will provide examples of the pathophysiological relevance of such interplay.

## Mechanistic aspects of the iNKT-myeloid cell crosstalk

The relevance of the interplay between iNKT cells and MPS populations can be defined as not univocal and linear (19), but dependent on several factors that can impact the reciprocal cell regulation *in vivo* such as: (i) the strength of cognate antigen/iTCR signal, co-stimulation and the maturation state of the mononuclear phagocytic cell; (ii) the iNKT cell subset involved in the interaction; (iii) the physiological vs. pathological status of the host. In this review, we add the tissue context as a fourth factor that has acquired relevance in recent years, as accumulating evidences are highlighting the importance of a fine-regulated crosstalk between iNKT cells and CD1d-expressing MPS in tissues for the biology of these cells.

The iNKT cell subsets involved in the interaction with MPS cells and the tissue context are strongly interconnected. Different tissues contain distinct composition of resident iNKT cell subsets, at least in mice (23–26). Based on the differential expression of three key transcription factors (PLZF, Tbet, RORγt) involved in the determination of specific effector phenotypes, mouse iNKT cells acquire T_H_1- (NKT1, PLZF^low^, Tbet^+^, RORγt^−^), T_H_2- (NKT2, PLZF^high^, Tbet^−^, RORγt^−^), and T_H_17-like (NKT17, PLZF^int^, Tbet^low^, RORγt^high^) cytokine profiles already upon thymic development. Recent reports suggest that this subsets definition for iNKT cells may not entirely represent the whole spectrum of effector functions displayed by these cells, as their effective cytokine production can sometimes deviate from the one expected from their transcription factor profile (27, 28). This suggests both that iNKT cells may undergo some sort of post-selection functional tuning, and the need for a more comprehensive phenotypical and functional analysis to define their effector profiles. Nevertheless, each known iNKT cell subset egresses from the thymus to survey different peripheral compartments. In C57BL/6 mice, NKT1 cells comprise the >95% of all hepatic iNKT cells, and are also predominant in the prostate, while NKT2 and NKT17 (29) are highly enriched in the intestine and lung mucosae, respectively. In secondary lymphoid organs, NKT1 and some NKT2 cells are contained in the spleen, while LNs harbor NKT1, low NKT2, and expanded NKT17 cells, with the notable exception of mesenteric LNs and Peyer's Patches, in which iNKT2 represent up to 40% of iNKT cells (24, 30). The adipose tissue contains a distinct IL-10 producing regulatory iNKT cell subset (NKT10) (25), which lacks PLZF but express the transcription factor E4BP4, and whose thymic vs. peripheral differentiation is currently unknown (31, 32). The relative frequency and tissue distribution of the iNKT cell subsets varies substantially between different mouse strains, likely correlating with the different dominant types of effector responses classically observed in each strain (24). iNKT cells are sessile cells that exhibit remarkable tissue-residency and limited recirculation, with the notable exception of those cells found in the peripheral blood (23, 25). Together, these characteristics confer iNKT cells a fundamental role in the tissue homeostasis and immune architecture: based on their main cytokine profiles they display in different tissues, iNKT cells modulate in different directions the effector response of the mononuclear phagocytic cells they interact with (33).

The pathophysiological status of the host can also influence iNKT cell distribution and subset balance, which may directly reflect on their communication with the MPS. For instance the relative composition of NKT1, NKT2, and NKT17 cells in a given tissue may be altered from physiology to pathology, as observed in prostate cancer progression (26), or in adipose tissue in lean and obese subjects (34, 35), impacting the quality of the resulting effector functions. This is an intriguing observation, which points to unanticipated effector plasticity and/or ability to migrate into different tissues of iNKT cells that would be relevant to understand.

A parallel aspect impinging substantially on the iNKT-myeloid cell crosstalk is represented by the functional plasticity characterizing the cells of the MPS, particularly monocytes/macrophages, which directly impact the pathophysiological status of the host. Indeed, monocytes are able to differentiate throughout a broad spectrum of effector phenotypes ranging from strongly pro-inflammatory and tissue damaging, to anti-inflammatory and tissue repairing profiles. For macrophages, this complex functional spectrum has been (over)simplified in the widely recognized paradigm of pro-inflammatory M1 and anti-inflammatory M2 populations, mirroring the T_H_1 and T_H_2 states of T cells (36), which represent the two functional extremes of the spectrum (37, 38). *In vivo*, however, macrophages appear often to exhibit mixed phenotypes, with a variable M1/M2 balance, which are modulate by the combination of molecular and cellular signals contained in the local microenvironment, implying a remarkable functional plasticity of this cell population (39).

The interplay between iNKT cells and MPS cells is mutual and embraces different aspects. iNKT cells depend for their functional education on CD1d^+^ mononuclear phagocytes (40, 41). At the same time, the maturation and polarization of DCs and monocytes is promoted by iNKT cells (42, 43). Several mechanisms could underlie this interplay, including CD1d engagement (44), cytokine production (45), CD40 ligation (46, 47), purinergic signaling (48, 49). iNKT cell-dependent signaling cues indeed direct the acquisition of either pro-inflammatory or anti-inflammatory effector phenotypes of myeloid cells (50–53). Based the above considerations, the outcome of the interconnections between iNKT cells and MPS cells in specific anatomical sites can thus be quite different.

## Secondary lymphoid organs

iNKT cell distribution in secondary lymphoid organs allows them to exert their “adjuvant” functions for both innate and adaptive immune response, culminating in the non-cognate or cognate help to B cell responses (54–58). In popliteal LNs at steady state, endogenous iNKT cells localize in the interfollicular region and medulla, but not in the T-cell-rich paracortex (59), whereas adoptively transferred iNKT cells are found in the paracortex (60), possibly reflecting the different methods used to detect the cells *in situ*. In the steady state spleen, both autochthonous and adoptively transferred iNKT cells are found widely distributed throughout the parenchyma, including B and T follicles in the white pulp, the marginal zone (MZ) and the red pulp (56, 61). This iNKT cell distribution at is substantially modified upon antigen-dependent activation. In the popliteal LNs, upon immunization of mice with particulated antigens formulated with the strong lipid agonist αGalCer, the adoptively transferred iNKT cells rapidly move from the paracortex to contact CD169^+^ macrophage lining the subcapsular sinus, which express CD1d and can present lymph-borne soluble antigens, resulting in a strong iNKT cell activation and secreting copious amounts of helper cytokines (60). In the spleen, the injection of soluble antigen formulated with αGalCer, or of pathogenic bacteria containing stimulatory glycolipids, results in the massive accumulation, within 8 h from administration, of splenic iNKT cells in the MZ, where the cells are activated upon contacting CD1d^+^DCs, and possibly also macrophages (61, 62). This iNKT cell re-distribution in secondary lymphoid organs as several functionally relevant consequences for the immune response: (1). It leads to the contact-dependent maturation of macrophages, which can limit potential pathogen spreading in secondary lymphoid organs, and of DCs, which relocate to T cell zones and promote downstream adaptive T and B cell responses, resulting in the so-called non-cognate iNKT cell help (42, 55, 59–62); (2). It elicits the secretion of copious amount of different helper cytokines by iNKT cells, which can stimulate innate and adaptive immune effectors throughout the LN and splenic parenchyma (60, 61); (3). It results in the acquisition of a follicular helper effector phenotype by iNKT cells (iNKT_FH_: Bcl6^high^CXCR5^high^PD-1^high^) (57, 63, 64), which can ultimately enter into the B cell follicles and help CD1d-expressing B cells presenting the same lipid antigen, providing the cognate iNKT cell help (56, 57, 65). In fact, although the interaction with CD1d-expressing B cells is fundamental to sustain the full iNKT_FH_ cell differentiation and functions (56), the upregulation of the follicular helper molecules by iNKT cells requires the recognition, in first place, of CD1d-expressing myelomonocytic APCs (56), most likely DCs (61), but possibly also CD169^+^ macrophages (60, 62). Interestingly, a recent study has gained new mechanistic insight into the critical role of the interaction between LN-resident CD1d^+^CD169^+^ macrophages and endogenous iNKT cells for the delivery of non-cognate help to B cells, activated in the course of influenza virus infection (28). Indeed, as early as 3 days upon influenza virus infection, iNKT cells are found in contact with “stressed” CD1d^+^CD169^+^ macrophages of the subcapsular sinus, in analogy with the results obtained by injecting particulated Ags containing αGalCer. There, iNKT cells become activated by CD169^+^ macrophages via CD1d-cognate Ag stimulation and secretion of IL-18, without acquiring iNKT_FH_ phenotype. This activation, in turn, induces rapid iNKT cell migration at the B cell follicular border and the secretion of copious IL-4, which is critical for the early phase of germinal center formation and anti-viral antibody responses. Expression of CD1d on macrophages, but not on B cells, is required to elicit IL-4 production by iNKT cells, and mice lacking macrophages or IL-4 develop fewer germinal centers and less influenza specific IgG1 than wild-type mice (28). It is intriguing that IL-18 release by sinus-lining LN macrophages, induced upon inflammasome-depending pathways activated by pathogen-related innate signals, can also elicit the rapid secretion of protective IFNγ by a network of innate and innate-like effectors that include iNKT cells, which are strategically prepositioned for pathogen sensing in secondary lymphoid organ (59).

Collectively, these evidences support a critical role for the iNKT/MPS cell axis in the lymphoid system to rapidly sense infections and damage, and immediately react by promoting local and systemic innate and adaptive immune responses.

## The liver

iNKT cells are the prominent T cell subset in the mouse liver, accounting for up to 30% of T lymphocytes. They are also present in the human liver, though at a 30 times lower frequency; nevertheless, both mouse and human hepatic iNKT cells undergo quantitative and qualitative dynamic changes in chronic inflammation/infections or cancer, suggesting active involvement in the pathological processes affecting the organ (66–68). iNKT cells crawl under basal conditions in liver sinusoids and arrest upon stimulation by cognate antigen recognition, or exposure to inflammatory cytokines IL-12 and/or IL-18 (69–71). The liver contains a rich monocytic/macrophage component, comprising Kupffer cells, which are self-maintaining, tissue-resident phagocytes originating from embryonic yolk sac, and monocyte-derived macrophages. Kupffer cells and macrophages adjust their phenotypes in response to local signals, which determine their ability to worsen or end liver injury. Both mononuclear phagocyte types express CD1d and can interact with liver iNKT cells, resulting in such functional reprogramming. A paradigm of this function has been highlighted by a recent study using a model of focal hepatic sterile thermal injury assessed by intravital microscopy, revealing that iNKT cells stop and are activated by IL-12, IL-18, and the recognition of self-stress lipid(s) presented by CD1d-expressing CCR2^high^Ly6C^high^ inflammatory monocytes migrating into the injured area. Interestingly, the self-lipid(s)+cytokine stimulation results in iNKT cell production of IL-4, but not IFN-γ, which promotes the transition from inflammatory to reparative (CCR2^low^Ly6C^low^ CX_3_CR1^high^) monocytes, ultimately leading to the healing of the injury by collagen deposition, wound revascularization and hepatocyte proliferation (53). Interestingly, human iNKT cells extracted from chronic HBV or HCV infected cirrhotic livers exhibit an IL-4^high^/IFNγ^low^ effector profile skewing, compared to iNKT cells from non-chronic viral infections (72). This is consistent with a pro-fibrotic and tissue repair activity that, in the context of a sustained liver injury, can lead to a pathological form of tissue regeneration. However, in mice, there are also examples of potent IFNγ production by iNKT cells elicited by Kupffer cells during *Borrelia burgdorferi* liver infections (71), or upon provoked inflammation and autoimmunity, which promotes M1 polarization of the attracted peritoneal macrophages and, in these cases, sustains tissue damage (73, 74). It is possible that the opposite effector responses dominated by IL-4 or IFNγ observed in sterile vs. infectious inflammation may be related also to the different antigenic potency of self vs. bacterial lipid antigens that activate hepatic iNKT cells. Hence, the iNKT cell/MPS crosstalk in the liver is multifaceted depending on the underlying pathological situation, the inflammatory cell type involved, and the weak vs. strong antigen stimulation. All these parameters, collectively, can lead to either tissue repair or damage through the reciprocal modulation of both iNKT cell and macrophage effector functions, even though liver resident iNKT cells are essentially all NKT1 at start. This observation suggests the possibility that the effector profile of liver iNKT cells may change in different pathological situation. As already discussed above, because iNKT cell are reported to be sessile and functionally rigid, an interesting question is whether, under pathological stimuli, liver iNKT cells may either be replaced by newly recruited ones that are endowed with different effector profiles, or undergo functional reprogramming in the organ, implying an unexpected functional plasticity that may apply also to other organs.

## The peritoneum and omentum

The peritoneum forms a unique microenvironment, which is formed by a thin mesenchymal membrane that lines the abdominal cavity and surrounds the visceral organs. The omentum is a large apron-like peritoneal fold that connects the spleen, pancreas, stomach and transverse colon (75), which encloses adipocytes and specialized compact structures (“milky spots”) containing macrophages, DCs, B cells, T cells and mast cells (76). The omental adipocytes expand in obesity, linking the omentum to the adipose tissue and the metabolic control (see below). The peritoneum is an active immune site, in which both branches of the immune system contribute to maintain homeostasis (77). In the murine peritoneum, iNKT cells are present in sizable quantity (78), while the human omentum is highly enriched in iNKT cells, at least 10 time more than any other human organ analyzed (34). Evidences suggest a close interplay between iNKT cells and the abundant population of CD1d^+^ macrophages found within the peritoneal membrane. iNKT cells negatively correlated with mouse survival in a model of abdominal sepsis (79, 80), while induction of abdominal sepsis in the peritoneum of iNKT cell-deficient (Jα18^−/−^) mice results in the reduction of Ly6C^low^ anti-inflammatory macrophages and decreased mortality compared to WT. The critical interplay between peritoneal iNKT cells and macrophages is further illustrated by a model of acute sterile inflammation, in which peritoneal macrophages phagocyte neutrophils (efferocytosis) leading to CD1d upregulation and IL-4 secretion. This process activates iNKT cells to produce large amounts of IL-4 that, in concert with the macrophage cytokine, sustains the M2-like polarization and the resolution of the inflammation (81). *In vivo*, peritoneal CD4^+^ iNKT cells are the major producers of IL-4 (81), suggesting the possibility that peritoneal iNKT cells are either NKT2, or acquire NKT2 phenotype upon stimulation.

## The adipose tissue

The immune system contained in adipose tissue (AT) is unique. Sizable quantities of innate-like T cells reside in the omentum and visceral AT of mice and humans (34, 82). Here, iNKT cells primarily interact with CD1d-expressing macrophages (83) and adipocytes (84) to maintain non-inflammatory conditions. In fact, the AT is a sophisticated sensor of metabolic alterations induced by dietary stimuli, and the status of AT-resident macrophages is of great importance for the physiological metabolic control at this site: pathological metabolic alterations associated with obesity results in profound modification of AT-macrophages, inducing pro-inflammatory (M1-like) functions and a consequent increase in local inflammation and insulin resistance (35, 85). Regulatory iNKT10 cells are selectively enriched in the AT and rapidly respond to stimulatory lipids presented by CD1d+ macrophages, or adipocytes, by secreting IL-4 that restrains M1 and promotes M2 polarization (25, 35, 83, 85, 86). However, a prolonged dysmetabolic state provokes down-regulation of CD1d on AT-M2 cells and their switch to an M1-like phenotype that, in turn, leads also to a pro-inflammatory shift of local iNKT cells (83, 87, 88), again suggesting a plasticity due to the migration of iNKT1 cells from other sites or a functional differentiation of local cells. The presence of iNKT cells in the AT, which is conserved between mouse and human, is crucial for the formation of fat-associated lymphoid clusters (FALC). FALC are non-capsulated structures in the adipose tissue that collect T_H_2-skewed immune cells, most notably ILC2 (89), which direct the polarization of B1 cells, eosinophils and M2 macrophages (90) in order to maintain the homeostasis in the tissue. FALC are absent in CD1d^−/−^ mice, while they can be induced following iNKT cell adoptive transfer in Rag2^−/−^, suggesting the critical dependency of these structures on iNKT cells (91). Under peritoneal inflammation, the activation of iNKT cells increases the formation of FALC, indirectly inducing the recruitment of beneficial anti-inflammatory myeloid cells and the resolution of inflammation.

## The gastro-intestinal system

In mice, under homeostatic conditions, gut infiltrating iNKT cells (small intestine and lamina propria) are NKT1 (>90%) or NKT17 (<10%) (92). NKT2 are barely detectable in the intestinal epithelium, although they represent up to 40% of iNKT cells of the mesenteric lymph node (LN) and of those infiltrating Peyer's Patches (24). The accumulation of iNKT cells in the small intestine and mesenteric LN has been confirmed also in humans (30). Intestinal macrophages maintain gut homeostasis through the clearance of enteric pathogens and the enforcement of the tolerance to food and microbiota antigens via the production of IL-10 (93, 94). Recent evidences point out that a heterogeneous CD11c^+^ myeloid population, which includes both DCs and macrophages, stimulate iNKT cells in the gastro-intestinal system (92), resulting in the control of the intestinal bacteria composition and compartmentalization, regulation of the IgA repertoire and induction of regulatory T cells within the gut. The recognition of microbial lipid products is pivotal for the physiology of intestinal iNKT cells (95–97). In this context, α-glycolipids that are recognized from iNKT cells can originate from the commensal flora (98, 99), or from the diet (100). Upon CD11c^+^ myeloid cell-activation, NKT17 and NKT2 cells in the mesenteric LN undergo rapid activation and expansion, suggesting a pathogenic role for these cells in ulcerative colitis (101).

## The lungs

In the steady state, the mouse lung contains iNKT cells that distribute predominantly in the vasculature, with a minority residing in the interstitium, which are belong to clearly distinct functional subsets. Whereas the majority of the lung-associated vasculature cells are NKT1, the lung interstitium contains the highest frequency of NKT17 in C57BL6 mice (>50%) (24, 102), which is consistent with their involvement in pathogen surveillance. Barrier epithelia (e.g., lungs, colon, skin, LN) produce elevated quantitates of IL-7 (103) which drives NKT17 survival and maintenance (104), thus creating a microenvironment favorable for the accumulation of these effector cells. The clearance of inhaled pathogen is the main feature of lung-resident (alveolar) macrophages (51). The iNKT cell-macrophages axis is once again critical in this context. In a model of viral-induced chronic airway inflammation, iNKT cells are directly recruiting and activating macrophages toward an anti-inflammatory, tissue remodeling M2-state (105). Increased amounts of iNKT cells and of IL-13 producing macrophages have been detected not only in mice, but also in patients with chronic obstructive pulmonary disease (COPD) (105, 106), supporting the involvement of the iNKT cell/macrophage crosstalk in the lung pathophysiology.

iNKT cells react also to a number of pathogens involved in airway infections, including *Sphingomonas capsulata* (107), *Mycobacterium tuberculosis* (108), *Pseudomonas aeruginosa* (109, 110), *Streptococcus pneumoniae* (111) and Influenza A virus (112, 113), via involvement of local mononuclear phagocytic cells, particularly macrophages. During *M. tuberculosis* infection in mice, iNKT cells are activated upon interaction with macrophages presenting mycobacterium-specific lipids (108) and help controlling the bacterial load via GM-CSF production (114), which may promote an inflammatory response that ultimately leads to bacterial clearance. A similar mechanism has been identified for *P. aeruginosa*, where iNKT cells stimulate increased phagocytic clearance of the bacteria in the lung by alveolar macrophages (109). Interestingly, in this context, iNKT cells have a stronger effect in controlling *P. aeruginosa* in BALB/c compared to C57BL6 mice (110). This difference can be explained by the different iNKT cell subsets that infiltrate the lungs of the two strains, as BALB/c mice contain an higher frequency of NKT1 subset compared to C57BL6 (24, 115). In the case of *S. Pneumonia* infection, intravital microscopy reveals that interstitial DCs present microbial glycolipids to the few adjacent iNKT cells, resulting in the neutrophil recruitment and CCL17 production. This promotes further iNKT cell migration from vasculature into acutely inflamed lung interstitium, where they assist DC activation and clearance of infection (116). This mechanism for acute inflammation seems conserved also in humans, as suggested by the human iNKT cell ability to drive *in vitro* the release inflammatory lipid mediators by monocyte-derived DCs, which can promote neutrophil recruitment and activation (48).

In addition to controlling bacterial infections, iNKT cells were also active in containing pulmonary infection influenza A virus. In this context, iNKT cells orchestrate anti-viral NK and CD8^+^ T cell responses (113, 117–119). iNKT cells promote virus control also by promoting differentiation into functional APC of lung-infiltrating immature myeloid derived cells, through CD40 engagement and CD1d cognate recognition (17), or by reducing pathogenic inflammatory monocytes (Ly6C^high^Ly6G^−^) via direct lysis (112), which correlates with better influenza outcome in iNKT cell-sufficient compared to insufficient mice.

## The tumor microenvironment

Cancer cells are embedded in the tumor microenvironment (TME), a complex and active milieu in which transformed and non-transformed cells dynamically interact in evolving networks that are continuously rearranged (120). The composition of the TME impinges heavily on the success of cancer therapy, and many studies underline the importance of targeting both the tumor and the supporting stroma for an effective and complete clearance of the malignancy (121). A substantial fraction of immune infiltrate of the TME is composed by tumor associated macrophages (TAMs) (122), which can encompass a spectrum of activation states largely affecting tumor progression, dissemination and response to therapy (36). Different stimuli present in the microenvironment can also rapidly trigger a number of diverse functions in macrophages, which range from the activation of potent pro-inflammatory M1-like responses, to the coordination of M2-like tissue remodeling and immunosuppression.

Despite their low numbers, iNKT cells are also components of the immune infiltrate present in both mouse and human tumors (26, 78, 123–125). Indeed, a growing body of evidences lends support to a critical role for these cells in modulating myelomonocytic cells in the tumor microenvironment. M1-oriented TAMs are generally beneficial for the control of tumors because by exerting critical functions such as antigen presentation, production of inflammatory cytokines and inhibition of angiogenesis (126, 127). By contrast, M2-like TAMs are detrimental, because they exert tumor-supporting, pro-angiogenic, pro-metastatic, and immunosuppressive activities (128). The first hints of iNKT cells interplay with TAMs come from the observation that these cells can kill in a CD1d-dependent manner transferred human macrophages infiltrating a xenograft model of human neuroblastoma in NOD/ SCID/IL-2Rγ-null (NSG) mice (129). The importance of the iNKT cell-TAM crosstalk is further strengthen in the same model, by showing that iNKT cells are recruited into tumor in a CCL20-dependent manner, but inhibited in their anti-tumor activity by macrophage-induced hypoxia (125). In the recent years, this dual relationship has been investigated more in detail. By using a mouse model of oncogene-induced pancreatic cancer, iNKT cells have been shown to have a preferential activity on M2-like macrophages, which are increased in CD1d^−/−^ pancreatic cancers (130). iNKT cells delay also the onset and organ infiltration of a mouse model of chronic lymphocytic leukemia (CLL), and their counts in blood independently predicts disease stability in CLL patients (78). iNKT cells remodel the supporting niche of CLL by controlling CD1d-expressing, patient-derived M2-like macrophage population, termed nurse-like cells (NLCs), which sustain leukemia cell survival (78, 131). The unique mechanism by which iNKT cells selectively modulate different subset of TAMs has been recently elucidated in a model of autochthonous prostate cancer (26). In this model, the presence of iNKT cells causally associates with the selective reduction of M2-like TAMs in the tumor microenvironment, leading to the control of tumor progression. Human prostate cancer aggressiveness correlates with reduced intra-tumoral iNKT cells and increased M2 macrophages, underscoring the clinical significance of this crosstalk (26). This selective restriction of M2 TAMs depends on the combinatorial engagement of CD40 and Fas on the surface of macrophages by tumor-infiltrating iNKT cells. Although both molecules are expressed to similar levels on either M1 or M2 TAM populations, the CD40L-CD40 pathway supported the survival only of the M1 population, likely by antagonizing the apoptotic death driven by Fas signaling. By contrast, CD40 expression does not protect M2 TAMs form FAS-dependent killing, suggesting a differential CD40 signaling between M1 and M2 macrophages. Remarkably, the ability to selectively eliminating pro-tumor M2 macrophages seems, thus far, unique to iNKT cells. Interestingly, however, a mouse transgenic model of colon adenocarcinoma represents an exception to this general mechanism. Here, iNKT cells support pre-malignant progression by suppressing T_H_1 responses, and promoting suppressive Treg and M2-polarization of TAMs, leading to increased intestinal adenomatous polyps formation (132). The dichotomous iNKT cell response in the two mouse tumor models may be related to changes undergoing in the different TMEs. In both healthy prostate and intestine tissues, the NKT1 and NKT17 are mostly represented. However, as tumor progresses, iNKT cells infiltrating intestinal polyps start to produce IL-10, while those in the prostate cancer setting remained T_H_1-oriented.

On the basis of the described evidences, it is tempting to speculate that tumor-infiltrating iNKT cells lead the immune reprogramming of the local TME by acting primarily on the MPS. This remodeling activity in the tumor context appears critically determined by the specific effector profile exhibited by iNKT cells in the target tissue in physiological conditions, before the development of the malignancy. It will be important to investigate such relationship in different cancers, particularly human ones, given also the interest to define possible different tissue resident iNKT cell subsets, as well as to harness these cells for cancer immunotherapy that exploits their unique potential to reprogram the tumor microenvironment.

## Challenges and future directions

Increasing evidence underscore the relevance of the iNKT cell/mononuclear phagocyte crosstalk in many different tissues, which may contribute to the induction, or the resolution, of tissue damage depending on the local effector phenotype exhibited by the two cell types interacting in the specific tissue. To this respect, iNKT cells are widely located in, non-lymphoid tissues in homeostatic conditions, at least in mice, which include (but are not limited to) the central nervous system (133), kidney (134, 135), eye (136), placenta (137), pancreas (138), and prostate (26). In all these sites, iNKT cells have the possibility of interacting, or have been suggested to interact, with tissue-resident MPS cells. However, the result of this crosstalk has not yet fully elucidated. In some cases, iNKT cells and resident mononuclear phagocytic cells show complementary functions. During acute kidney injury, iNKT cells alleviate the induced damages in different models (135, 139–141). Interestingly, these pathological conditions are highly dependent on renal macrophages, that switch between M1 and M2 phenotype during the acute or the tissue-repair phase (142), or on immature DCs (135), suggesting a link with NKT1 or NKT2 cells. In the eye, iNKT cells contribute to the natural tolerance occurring at this site (136) by cross-talking with T cells, neutrophils and macrophages (143). During reproduction, iNKT cells are present in the placenta, the interface between the mother and the fetus (144), and play role in orchestrating the immune response during infections occurring during pregnancy (145). Considering that iNKT cells consistently infiltrate the placenta also in healthy pregnancies (137), it is reasonable to hypothesize that their role is not limited to pathological conditions but they constantly support the reproduction process, for instance maintaining the status of tolerance induced by IL-10 producing macrophages (146). In the pancreas, iNKT cells promote an innate response against LCMV, by enhancing the local recruitment of pDCs and stimulating their production of anti-viral type I IFNs via OX40-OX40L interaction (138). However, CD1d expression by pDCs is not required for this interaction, suggesting a different, yet undefined, mechanism from those described in other tissues. Given the long standing implication of iNKT cells in the control of Type 1 Diabetes, it would be interesting to assess whether and by what mechanisms these cells may modulate MPS cells in the pancreas (147).

Some of the signals controlling the meeting between the two cell types have been defined, however this remains an open area to explore. Mouse and human iNKT cells express chemokine receptor pattern typical of trafficking toward inflammation sites (148–151) which overlaps at least in part with that of monocytes, supporting a cooperative engagement at inflammatory sites. Indeed, it has been shown that following *B. burgdorferi* infection Kupffer cells induce CXCR3-dependent clustering of iNKT cells (71), while CXCR6 drives homeostatic iNKT localization in liver sinusoids (69). A final big gap in knowledge concerns details on the presence of different iNKT cell subsets in different human tissues and their possible interaction with MPS cells. Correlative studies suggest undergoing crosstalk between the two cell types also in human tissues, although direct evidence is substantially lacking. A more precise definition of these mechanisms, focusing in particular on the human system in physiology and pathology, should drive future studies.

## Concluding remarks

The crosstalk between iNKT cells and cells of the MPS has a critical role in both physiological and pathological conditions. The outcome of this interaction is highly dependent on the tissue where it occurs and can be either beneficial or detrimental for the host. Harnessing this crosstalk has potential therapeutic relevance in different pathologies, from cancer to infections, chronic inflammatory diseases or metabolic disorders, as well as to improve vaccine formulation.

## Author contributions

All authors listed have made a substantial, direct and intellectual contribution to the work, and approved it for publication.

### Conflict of interest statement

The authors declare that the research was conducted in the absence of any commercial or financial relationships that could be construed as a potential conflict of interest.
